# Low Cluster of Differentiation 4+ T Cell Count in People Living with HIV/AIDS Undergoing Antiretroviral Therapy Visiting a Reference Laboratory: A Descriptive Cross-sectional Study

**DOI:** 10.31729/jnma.6062

**Published:** 2021-05-31

**Authors:** Samikshya Kandel, Sundar Khadka, Mahesh Lamsal, Bimlesh Jha, Sunil Paudyal, Swotantra Gautam, Sagar Poudel, Mukunda Sharma, Jeena Amatya

**Affiliations:** 1 Nepal Academy of Science and Technology, Khumaltar, Lalitpur, Nepal; 2 National Public Health Laboratory, Teku, Kathmandu, Nepal; 3 Department of Biotechnology, Tribhuwan University, Kirtipur, Kathmnadu, Nepal; 4 B.P. Koirala Institute of Health Sciences, Dharan, Nepal; 5 All India Institute of Medical Sciences, New Delhi, India; 6 St. Xavier's College, Maitighar, Kathmandu, Nepal

**Keywords:** *acquired immunodeficiency syndrome*, *cluster of differentiation 4+ lymphocyte count*, *human immunodeficiency virus*

## Abstract

**Introduction:**

Human Immunodeficiency Virus is a lentivirus that causes human immunodeficiency virus infection and over time, acquired immunodeficiency syndrome. Cluster of Differentiation 4+ T cell count of people living with this infection play a vital role to determine infection progression and necessary treatment changes. This study was conducted to find out the prevalence of low Cluster of Differentiation 4+ T Cell Count in the People Living with human immunodefieciency virus/ acquired immunodeficiency syndrome.

**Methods:**

A descriptive cross-sectional study was conducted between June to August 2018 in the Human Immunodeficiency virus and Hepatitis Reference Unit of National Public Health Laboratory, Ministry of Health and Population Teku. Ethical approval was taken (Reference Number 2912) and a total of 550 seropositive cases of Human Immunodeficiency Virus-1 undergoing antiretroviral therapy were studied. Convenient sampling technique was used. Data was analysed by Statistical Package for the Social Sciences.

**Results:**

Seventeen (3.1%) of patients had Cluster of Differentiation 4+ T cell counts below 100 cells/ mm^3^ of blood. The mean Cluster of Differentiation 4+ T cell count was 509.3 cells/mm^3^ of blood. Of the total samples, 280 (50.9%) were males, 268 (48.7%) were females, and the rest 2 (0.4%) were of other gender.

**Conclusions:**

Majority of people living with human immunodeficiency virus/ acquired immunodeficiency syndrome were found immune-competent.

## INTRODUCTION

Human Immunodeficiency Virus (HIV) is a lentivirus (a subgroup of retrovirus) that cause HIV infection and over time, Acquired Immunodeficiency Syndrome (AIDS).^1^ This varies with the gender, occupation, injectable drug usage, among the people living with HIV/AIDS (PLHIV) in different stages of infection etc.^2^

National algorithm by the National Centre for AIDS and Sexually Transmitted Diseases Control have been working for HIV diagnosis, treatment and expansion of testing sites with ART service to PLHIVs.^3^ Determination of CD4+ T cell number is essential in proper treatment and prophylaxis since CD4+ T cell is the marker of infection.^4^

This study was undertaken to find out the prevalence of low CD4+ T cell count of PLWHA.

## METHODS

A descriptive cross-sectional study was conducted between June to August 2018 in the HIV and Hepatitis Reference Unit of National Public Health Laboratory (NPHL), Department of Health Services Ministry of Health and Population Teku, Kathmandu, Nepal. Ethical approval of the study was obtained from the Nepal Health Research Council (Ref No. 2912) before the commencement of the research. PLHIV under antiretroviral therapy were included . New cases or those not using antiretroviral therapy were excluded from the research. The convenient sampling technique was used and the sample size of this study was calculated as,


$$\begin{array}{ll} \text{n} & \text{= }{\text{Z}}^{\text{2}}\times \text{p}\times \text{q }/{\text{e}}^{\text{2}}\\ & \text{= }1.96^{\text{2}}\times 0.5\times (1-0.5)/0.05^{\text{2}}\\ & \text{= }384.16\\ & =385 \end{array}$$

Where,

n = minimum required sample size
Z = 1.96 at 95% Confidence Interval (CI)
p = prevalence taken as 50% for maximum sample size
q = 1-p
e = margin of error, 5%

Taking a non-response rate of 10%, the required sample size was 424. However. a total of 550 confirmed HIV positive cases under antiretroviral therapy treatment were taken as sample for the study. A convenient sampling technique was used and inclusion criteria was those PLHIV under antiretroviral therapy and the new cases or those not using antiretroviral therapy was the exclusion criteria for the research.

Five mL blood sample of HIV positive patient visiting reference laboratory was collected in Ethylenediamine tetra acetic acid (EDTA) vial by trained registered technical laboratory personnel only after counseling. The sample volume was divided into; 2mL venipuncture blood in EDTA vial for CD4+ T cell counting. All the laboratory works were performed in the NPHL equipped Biosafety label hood (Type 2A).

CD3\CD4\CD45 cell number was determined using whole EDTA blood sample by fluorescence-activated cell sorting technique using BD FACS CALIBUR. The standard Protocol for CD4+ T cell testing was followed as stated by BD Tritest CD3FITC\CD4PE\CD45 PerCP Catalogue. If the delay was anticipated, the blood sample for CD4+ T cell count was kept at room temperature and processed within 72 hours.

As an internal quality control, venipuncture blood sample a healthy person (person not infected with HIV), was taken in EDTA vial and tested in duplet.

Data and information were collected based on tests performed in the laboratory and structured validated questionnaire after taking consent from the participants of the study. These observations were maintained in the computer database in MS-EXCEL and analyzed by using Statistical Package for the Social Sciences (version 16.0). The University Grants Commission, Nepal, financially supported this study.

## RESULTS

Out of 550 samples, 17 (3.1%) of them were found to have CD4+ T cell count less than 100 cells\mm^3^, which is considered as one of the important parameters to define the AIDS stage of infection. The mean Cluster of Differentiation 4+ T cell count was 509.3 cells/mm^3^ of blood (Table 1).

**Table 1:** CD4 T cell count.

CD4+ T cell count	n (%)
<100	17 (3.1)
101-200	45 (8.2)
201-349	95 (17.3)
350-500	98 (17.8)
>500	295 (53.6)

Two hundred eighty (50.1%) were males, 268 (48.7%) were females, and 2 (0.4%) respondents of other sex groups were reported. The highest number of PLHIVs was represented by the age group between 30-39 years of age with average age value 36.4 years (Table 2).

**Table 2:** Classification of PLHIVs based on demographic and laboratory study parameters.

Demographic Profile		n (%)
**Sex**	Male	280 (50.1)
	Female	268 (48.7)
	Others	2 (0.4)
**Age group (years)**	0-9	11 (2)
	10-19	50 (9.1)
	20-29	59 (10.7)
	30-39	196 (35.6)
	40-49	163 (29.7)
	≥50	71 (12.9)

**Figure 1.**
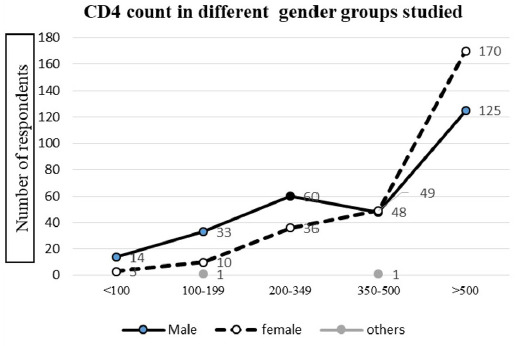
Distribution of CD4+ cell count in different genders of PLHIV

Distribution of immunological status of PLH is depictedas follows (Figure 1).

## DISCUSSION

This study highlights the status of CD4+ T cell count in PLWHA. The ratio of male to female was found to be 1.04. Similar finding was reported by Ojha, et al. 1.03 in a research conducted in 2016 in Nepal.^5^ The highest number of respondents was of age group between 30-39 years, which was 196 (35.6%). This might be due to the reason that population groups of this age are vigorously active in livelihood matters with high mobility within and outside nations for different purposes includes foreign employment, business, and other various reasons including sexually active age.^6^ A similar type of finding was reported in the research of Oluoch, et al. in Kenya and Ionita, et al. in 2017.^7^ In contrast, only 9.5% population was above 50 years of age in a study conducted by Oluoch^8^ which is 12.9% in our research.

The PLHIV participating in the research from Province 3 outnumbered other location, which might be due to the several reasons like, decentralized CD4+ T cell in a different location around the nation in 74 various Health institutions, in government and private levels (3) because of which a very few PLHIVs outside the valley, visited the lab. Those people from other provinces who participated in the research visiting the laboratory might be due to the stigma issue in their place since social stigma is still one of the major issues to overcome among PLHIV.^9^

With respect to immunological status based on CD4+ T cell count in five classes, <100, 100-200, 201-350, 351-500, >500 cells\mm^3^; more than half of the patients had CD4+ T cell number greater than 500 cells\mm^3^ which were immunologically competent based on the CD4+ T cell count. Apart from this, 61 patients had CD4+ T cell count less than 200\mm^3^, which are at high risk of attack by other opportunistic pathogens^10^ as recommended by WHO.^11^ Mean CD4+ T cell count was found to be 590. 3, which was found to be 501 (95% CI), in research by Ojha, et al. in 2016. In some cases, the patient having immune-competent CD4+ T cell count was also attacked by the opportunistic pathogen, which suggests that CD4+ T cell is not a single immune marker and one can solely rely on to determine the status of health of PLHIV.^12^

According to WHO guidelines, patients having CD4+ T count equal to or less than 350 cells\mm^3^ are recommended to start ART. In case of lack of improvement of health, regarding the reconstitution of CD4+ T cell count, even after initiation of ART, i.e. persistent CD4+ T cell count less than 200 cells\mm^3^ is referred to as immunological failure.^13^ In this study, eight patients CD4+ T cell less than 200\mm^3^, which means they are at great risk of confections.

HIV has asymptomatic pathogenicity after the window period in which the patient remains normal but is prone to transmit the infection indeed seems to have a healthy life. So, it is recommended among the risk group to get tested and initiate ART as soon as possible. So far, “Test and Treat” has been the topmost priority of practice for the identification and management of HIV infection, however, it might not include those who will be able to suppress the viral load naturally without the aid of ART.

The overall immunological profile of the study population was immune-competent and the number of people initiating ART seems to be increasing recently, which is a positive indication. So, proper consideration of clinical parameters and immunological aspects would stand out to be crucial for regular monitoring of disease progression in HIV infection in countries like Nepal.

## CONCLUSIONS

Thus, the overall immunological status of PLHIV included in the research was found to be immune-competent. A few cases were critical with CD4+ T cells count below 100cells\mm^3^ for more than 3 years. The findings of this study could be useful to find cases where immune-reconstitution is not successful even after the initiation of ART.
